# Temporal Validation of a Plasma Diagnosis Approach for Early Alzheimer Disease Diagnosis in a Cognitive Disorder Unit

**DOI:** 10.3390/jpm15100475

**Published:** 2025-10-02

**Authors:** Aleix Martí-Navia, Alejandro López, Lourdes Álvarez-Sánchez, Laura Ferré-González, Angel Balaguer, Miguel Baquero, Consuelo Cháfer-Pericás

**Affiliations:** 1 Alzheimer’s Disease Research Group, Instituto de Investigación Sanitaria La Fe, Avda de Fernando Abril Martorell, 106, 46026 Valencia, Spain; aleix_marti@iislafe.es (A.M.-N.); alex_lopez@iislafe.es (A.L.); lourdes_alvarez@iislafe.es (L.Á.-S.); laufegon@alumni.uv.es (L.F.-G.); baquero_miq@gva.es (M.B.); 2 Plataforma de Big Data, IA y Bioestadística, Instituto de Investigación Sanitaria La Fe, 46026 Valencia, Spain; angel_balaguer@iislafe.es; 3 Division of Neurology, Hospital Universitari I Politècnic La Fe, 46026 Valencia, Spain

**Keywords:** Alzheimer disease, plasma, biomarkers, diagnosis, validation, clinical unit

## Abstract

**Background**: Nowadays, there is a lack of reliable and minimally invasive diagnosis methods for the early detection of Alzheimer’s disease. The development and validation of such tools could significantly reduce the dependence on more invasive and costly confirmatory procedures, such as cerebrospinal fluid biomarkers analysis and neuroimaging techniques. **Objectives**: The main objective of this study is to validate the clinical performance of a previously developed diagnosis model based on plasma biomarkers from patients in a cognitive disorder unit. **Methods**: A new cohort of patients was recruited from the same cognitive disorder unit (n = 93). Specifically, demographic data (gender, age, and educational level), plasma biomarkers levels, and genotype (glial fibrillary acidic protein, phosphorylated Tau 181, amyloid-beta42/amyloid-beta40, apolipoprotein E) were collected to evaluate both approaches of the previous diagnosis model (one-cut-off, two-cut-off). **Results**: The one-cut-off approach showed a sensitivity of 74.3%, a specificity of 89.5%, and an area under the curve of 0.888, while the values for the two-cut-off approach were sensitivity of 66.7%, specificity of 99.9%, and area under the curve of 0.867. **Conclusions**: A multivariate diagnostic tool was temporally validated for implementation in a clinical unit. In fact, satisfactory results were obtained from both approaches (one-cut-off, two-cut-offs), but the two cut-offs approach was more consistent in correctly identifying non-Alzheimer’s disease cases, allowing us to identify a large number of cases with high specificity.

## 1. Introduction

Alzheimer’s Disease (AD), the leading cause of dementia, is characterized by a progressive cognitive decline [1]. The rising global prevalence of AD, together with the recent approval of disease-modifying therapies (e.g., lecanemab, donanemab) [2,3], underscore the urgent need for accurate and early diagnostic methods. The pathophysiological hallmarks of AD involve the extracellular accumulation of amyloid-beta (Aβ) as plaques, intracellular accumulation of phosphorylated Tau as neurofibrillary tangles, and elevated levels of glial fibrillary acidic protein (GFAP) surrounding Aβ plaques [4]. Currently, AD diagnosis relies on cerebrospinal fluid (CSF) biomarkers [5] or positron emission tomography (PET) amyloid [6]. However, these approaches are invasive and expensive, limiting their feasibility for the general population. Consequently, recent research has focused on the identification and quantification of plasma biomarkers [7,8]. Nonetheless, clinical validation of these biomarkers remains essential to establish a reliable, early, and AD-specific diagnosis, suitable for routine implementation in cognitive impairment units.

Regarding AD diagnosis tools, recent works focused on the evaluation of plasma biomarkers (total-Tau (t-Tau), phosphorylated Tau (p-Tau181, p-Tau217), neurofilament light (NfL), GFAP, Aβ42, Aβ40) [9,10]. Several investigations have incorporated additional variables (e.g., genetics, demographics, neuroimaging, cognitive tests) and used different cohorts (Multimodal Interventions to Delay Dementia and Disability in rural China, Shandong Yanggu Study of Aging and Dementia [11], Alzheimer’s Disease Cardiff Cohort [12], Alzheimer’s Disease Neuroimaging Initiative (ADNI) [13]). Furthermore, genetic variables have been examined, encompassing apolipoprotein E (APOE), bridging integrator 1 (BIN1), CD2-associated protein (CD2AP), clusterin (CLU), and inositol polyphosphate-5-phosphatase D (INPP5D) [14]. In addition, a previous study used plasma biomarkers, together with magnetic resonance imaging (MRI) variables, genetics, lifestyle, and cardiovascular factors to improve prediction in patients [15]. The combination of different types of variables has led to the development of potential diagnosis models. However, few of them have been validated.

Concerning clinical validation of potential diagnosis tools, Cano et al. used three different cohorts (modeling, testing, and validation) to evaluate plasma p-Tau181 levels, distinguishing between individuals with prodromal mild cognitive impairment (MCI) and those with non-prodromal MCI [16]. Other studies used their own cohorts (e.g., Memory Aging and Cognition Centre (MACC) Harmonisation Cohort, Butler Alzheimer’s Prevention Registry) [17,18]. In addition, plasma p-Tau217 provided satisfactory results to predict Aβ status in a cohort from Butler Hospital Memory & Aging Program (MAP) (AUC 0.88), with similar performance in the BioFINDER-2 training cohort (AUC  =  0.99) [18]. A few studies from the literature were based on temporal validation, which is the simplest form of external validation [19]. Actually, Qin et al. used the ADNI cohort, with a model development set and a temporal validation set; the AUCs obtained for each set were similar [20]. Another work was based on a multicenter hospital retrospective study, and it used three different cohorts (derivation sample, temporal external validation, geographical external validation), providing satisfactory results (sensitivity of 92.5%, specificity of 89.5%, AUC = 0.91) in the temporal validation cohort [21].

This paper aims to evaluate the ability of a previously developed screening model based on plasma biomarkers to identify patients at risk of AD [10]. As specific objectives, two model approaches (one-cut-off, two-cut-offs) will be validated on a cohort of patients recruited in the same cognitive impairment unit in a subsequent time interval; patients will be stratified into different AD risk groups (high, medium, low), by means of this validated screening method, which could be very useful in a cognitive disorder unit in order to limit confirmatory tests only for uncertain cases, as well as to plan personalized follow-up and therapeutic strategies.

## 2. Methodology

### 2.1. The Derivation Cohort and the Diagnosis Approach

**Figure 1:**
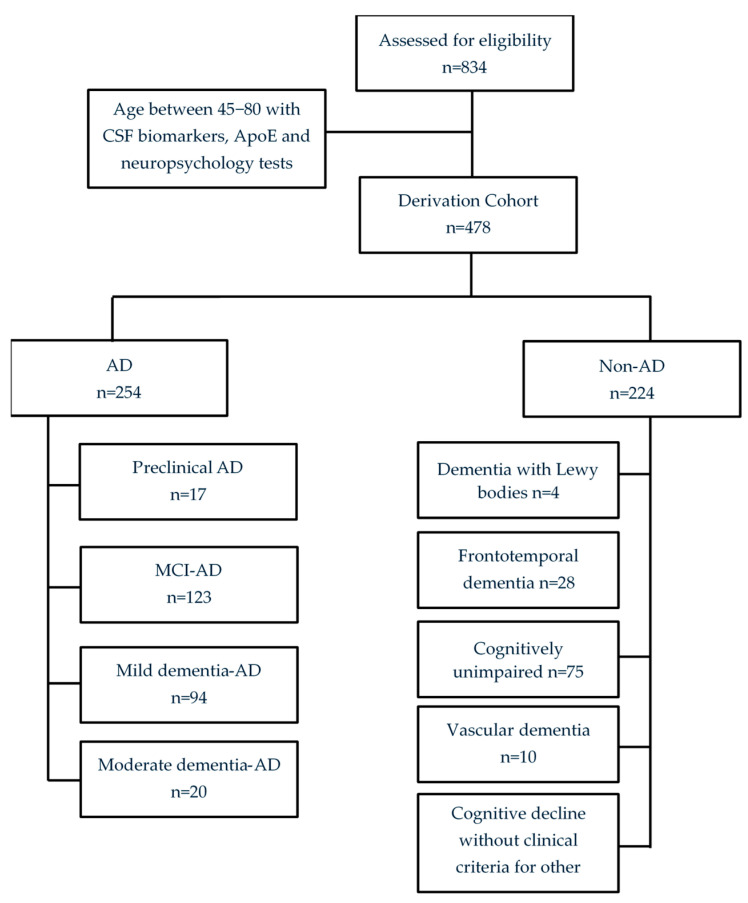
Flow chart from the derivation cohort.

The derivation cohort consists of 478 patients enrolled in the project from 2019 to 2023 in the Cognitive Impairment Unit of Hospital Universitari i Politècnic La Fe (Valencia, Spain; see Figure 1).

**Figure 2:**
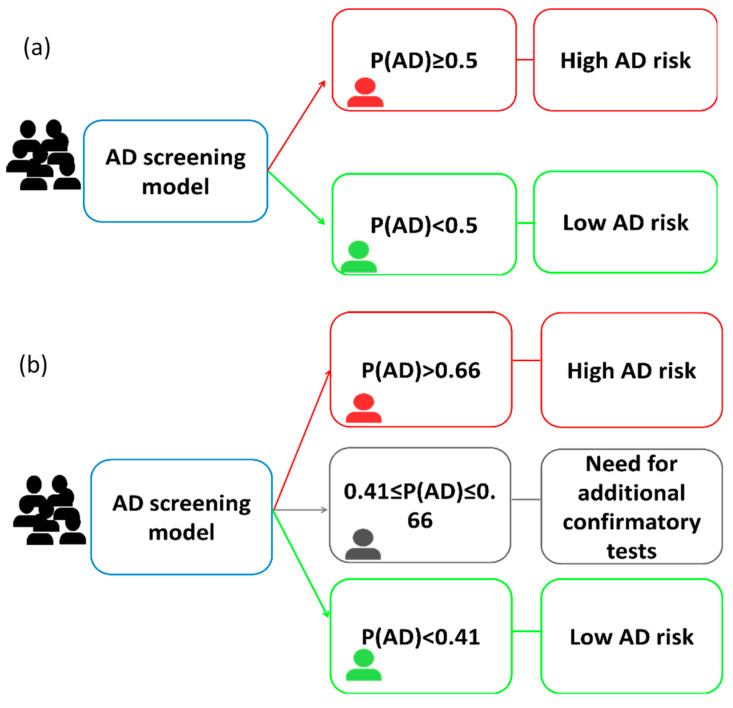
Scheme of the potential screening method based on: (**a**) one-cut-off approach, and (**b**) two-cut-off approach [10].

This cohort was used to develop an AD diagnosis model, described in a previous study [10]. Briefly, it was developed from specific plasma biomarkers, and some cut-offs were established to identify those patients at risk of AD (see Figure 2). The equation used to calculate the probability of developing AD is as follows:

$$P\left(AD=yes\right)=\frac{1}{1+e^{-\left(\beta_{0}+\beta_{1}\times pTau181+\beta_{2}\times AB42/AB40+\beta_{3}\times GFAP+\beta_{4}\times APOE1+\beta_{5}\times APOE2+\beta_{6}\times APOE3+\beta_{7}\times APOE4+\beta_{8}\times Age\right)}}$$

### 2.2. The Validation Cohort

The validation cohort of this observational pilot study consists of 93 patients enrolled in the project from 2023 to 2024 in the Cognitive Impairment Unit of Hospital Universitari i Politècnic La Fe (Valencia, Spain). They showed memory complaints. Their diagnosis was based on CSF Aβ42/Aβ40 levels and neuropsychological tests, according to standard criteria of the National Institute of Aging-Alzheimer’s Association [22,23,24].

The inclusion criteria were the same as those defined for the previous study [10]. Briefly, participants were age ≥ 50 years, provided informed consent, had available CSF biomarkers, and underwent neuropsychological evaluation; and the exclusion criteria were withdrawal of informed consent, other neurological disorders or serious mental disorder, or severe dementia.

CSF biomarkers levels were determined by chemiluminescence (CLIA) immunoassay (Lumipulse^®^ Fujirebio, Tokyo, Japan), and the cut-off point for Aβ42/Aβ40 was <0.069 for AD. Neuropsychological evaluation was performed by qualified neuropsychologists and it consisted of clinical dementia rating (CDR) [25]; Mini-Mental State Examination (MMSE) [26,27]; Repeatable Battery for the Assessment of Neuropsychological Status (RBANS) [28,29]; Functional Activities Questionnaire (FAQ) [30]; Alzheimer’s Disease Cooperative Study—Activities of Daily Living (ADCS-ADL) [31]; and Geriatric Depression Scale (GDS) [32].

**Figure 3:**
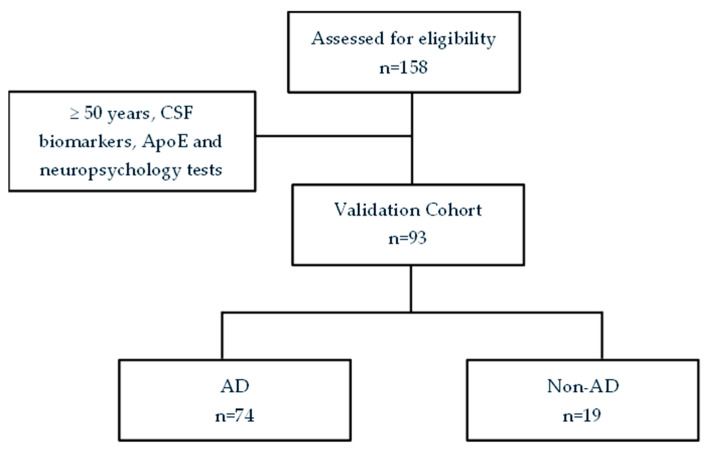
Flow chart from the validation cohort.

Patients were classified into the AD group (n = 74, CSF Aβ42/Aβ40 < 0.069) [33] and the non-AD group (n = 19, CSF Aβ42/Aβ40 > 0.069; see Figure 3). The AD group included patients without cognitive impairment, with MCI, and with mild dementia. The non-AD group included patients without cognitive impairment, with other dementias (frontotemporal dementia, dementia with Lewy bodies, vascular dementia), and with cognitive decline that did not meet clinical criteria for other specific pathologies.

The study was conducted in accordance with the Declaration of Helsinki, and it was approved by the Ethics Committee from Instituto de Investigación Sanitaria La Fe (Valencia, Spain) (reference number: 2022-990-1; date: 8 February 2023). All participants signed informed consent prior to their recruitment.

### 2.3. Plasma Samples Collection and Determination of Biomarkers

As in the previous study [10], blood samples were obtained using a tube containing EDTA, the samples were centrifuged (15 min, 1160 g at room temperature), and the plasma fraction was separated. These plasma samples were collected on the same day as the CSF sampling. The plasma samples were stored at −80 °C until analysis. At the time of sample collection, none of the participants were undergoing any pharmacological AD treatment.

A platform based on SIMOA^®^ technology (Quanterix SR-X^TM^ equipment (Billerica, MA, USA)), was used to determine the plasma biomarkers (p-Tau181, GFAP, Aβ42/Aβ40). The corresponding kits were purchased from Quanterix (Billerica, MA, USA).

### 2.4. Apolipoprotein E Genotyping

Genotyping of Apolipoprotein E (ApoE) was carried out from blood DNA. This analysis was performed using PCR and Light-Mix^®^ Kit ApoE C112R R158C from Roche Diagnostics. All the participants signed the corresponding informed consent.

### 2.5. Statistical Analysis

The sample size was estimated, assuming a simple random sampling design. The expected accuracy was set at 0.84 (cross-validated accuracy) to achieve a 95% confidence interval with a margin of error of ±7.5%. For this, the R software version 4.5.0, package samplesize4surveys version 4.1.1., and ss4p function were used.

Numerical variables were expressed as the median and interquartile range (IQR), and the Mann–Whitney test was used to compare differences between groups; categorical variables were expressed as total number and relative percentage, and the Chi-Square test was used to compare differences between groups. Correlations between numerical variables were evaluated using the Spearman test with the rank correlation coefficients (rho). For all the analyses, statistical significance was established as *p*-value < 0.05.

For the temporal validation of AD predictive model, AD probability was calculated using plasma variables (Aβ42/Aβ40, p-Tau181, GFAP), ApoE genotype, and age. Two classification approaches were employed. For the one-cut-off approach, patients with probabilities P(AD) ≥ 0.5 were classified as AD patients, while P(AD) < 0.5 were classified as non-AD. For the two-cut-off approach, patients with P(AD) ≥ 0.66 were classified as AD patients, while P(AD) < 0.41 were classified as non-AD, and patients with P(AD) between 0.41 and 0.66 were classified as uncertain cases, needing an additional test. The performance of the validated model was evaluated by calculating AUC, sensitivity, specificity, positive predictive value (PPV), negative predictive value (NPV) and accuracy. The SPSS v23 software (Chicago, IL, USA) was used in all the analyses.

## 3. Results

### 3.1. Participants Description

The demographic and clinical description of the participants groups is summarized in Table 1. As can be seen, statistically significant differences were found for age, ApoE genotype, CSF biomarkers, AT classification, and some neuropsychological tests (CDR_GS, RBANS_DM, GDS). The sample size estimation showed that a minimum of 92 subjects was required to achieve a 95% confidence interval with a margin of error of ±7.5% for the expected accuracy (0.84).

### 3.2. Plasma Biomarkers

Plasma biomarkers levels found for each participants group are summarized in Table 2. As can be seen, statistically significant differences were obtained for p-Tau181 and GFAP, with higher levels in the AD group, and for Aβ42/Aβ40, with lower levels in the AD group.

### 3.3. CSF, Relationship Between Plasma Biomarkers Levels and Clinical Variables

Bivariate correlations between plasma biomarkers and clinical variables (neuropsychological tests, CSF biomarkers) showed some statistically significant relationships (see Table 3).

### 3.4. Temporal Validation of a Previous AD Diagnosis Model

#### 3.4.1. One-Cut-Off Approach

**Figure 4:**
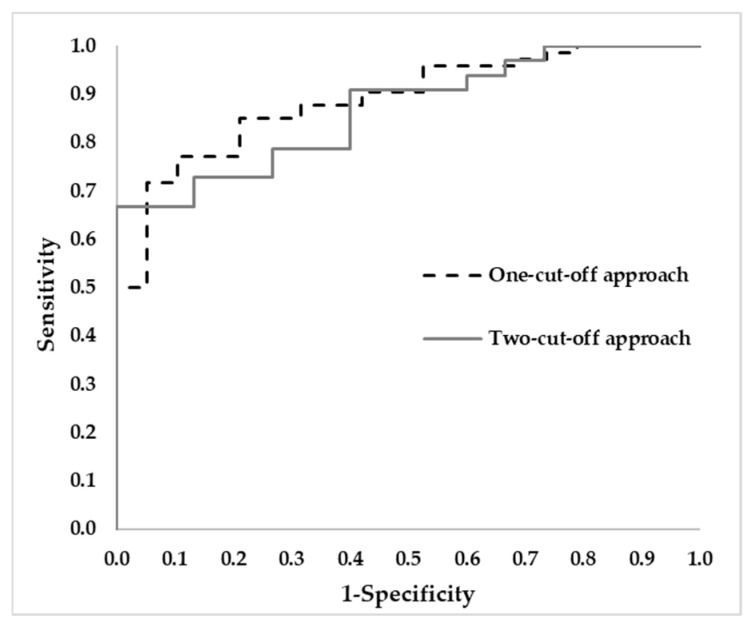
ROC Curves for both approaches of AD diagnosis under evaluation (one-cut-off, two-cut-off).

From the one-cut-off approach [10], the P(AD) was estimated for each patient, and they were classified as AD (n = 57), and non-AD (n = 36). These results were compared to the reference diagnosis based on CSF Aβ42/Aβ40 levels. Diagnostic performance metrics are shown in Table 4, and the ROC curve is depicted in Figure 4, with AUC 0.888

#### 3.4.2. Two-Cut-Off Approach

From the two-cut-off approach [10], patients were classified as AD (n= 22), non-AD (n= 26), and uncertain (n= 45). Comparing these results with the reference classification based on CSF biomarkers, the diagnosis indexes were calculated (see Table 4). In Figure 4 the ROC curve that was obtained is depicted, with AUC 0.867.

#### 3.4.3. Comparison of Diagnosis Tools

Both approaches showed similar performance, with similar values in terms of AUC, PPV, NPV, and accuracy. However, the two-cut-off approach showed higher specificity and lower sensitivity compared to the one-cut-off approach (see Table 4).

**Figure 5:**
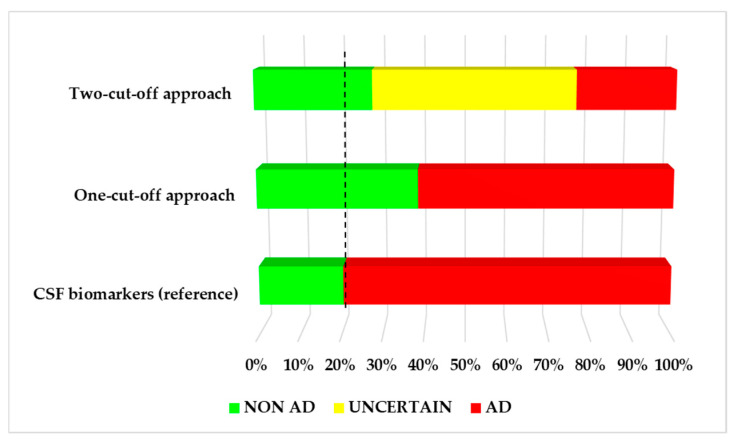
Risk stratification for the one-cut-off and the two-cut-off approaches, in comparison with the reference CSF classification.

Figure 5 illustrates the AD risk stratification for each approach (one-cut-off, two-cut-offs), as well as the reference CSF classification. As can be seen, CSF analysis classified 80% (n = 74) of the patients as AD, and 20% (n = 19) of them as non-AD. The one-cut-off approach classified the patients as AD (61%; n = 57) and non-AD (39%; n = 36). In this case, 19% of the cases identified as non-AD showed inconsistency with the reference classification (CSF biomarkers). The two-cut-off approach classified the patients as AD (24%, n = 22), non-AD (28%, n = 26), and 48% as uncertain cases (n = 45). In this case, 8% of the cases identified as non-AD showed a discrepancy with the reference classification based on CSF Aβ42/Aβ40 levels.

## 4. Discussion

The present study aims to evaluate the ability of a previously developed screening model based on plasma biomarkers to identify patients at risk of AD. This validation is required to be implemented as a screening method in clinical routine. To achieve this validation, patients were classified as AD vs. non-AD according to CSF Aβ42/Aβ40, which represents an established early biomarker of AD. Then, a simple and specific diagnosis model based on three plasma biomarkers (p-Tau181, Aβ42/Aβ40, GFAP) and ApoE genotype was validated in a cohort from the same cognitive impairment unit. Specifically, two approaches (one-cut-off, two-cut-off) were evaluated, showing satisfactory performance to identify patients at risk of developing AD.

Temporal validation assesses the performance of a diagnosis model in a subsequent cohort of patients recruited from the same cognitive disorders’ unit (HUiPLaFe). Several studies in the literature used temporal validation cohorts with one-cut-off points (e.g., MGH cohort, Boston cohort, ADNI…) [20,21,35,36,37,38]. In general, they used plasma biomarkers (p-Tau181…) or combinations of biomarkers (e.g., Aβ PET, CSF Aβ42/Aβ40, CSF p-Tau181/Aβ42). Across these studies, reported sensitivity values ranged from 74.1% to 93.3%, specificity values from 69.6% to 100%, and AUC values from 0.778 to 0.948. These values showed satisfactory performance for the evaluated diagnosis models, but they did not include plasma p-Tau217 [35,36,37,38]. Other studies did not count with CSF or PET confirmations, did not include healthy controls, or their sample size was very small [36,37]. In comparison with the present study, similar results were obtained for specificity and AUC, showing that the diagnosis approach was satisfactorily validated. In general, this method could be a potential diagnostic approach to replace CSF biomarkers in the detection of AD. Specifically, plasma p-Tau217 and p-Tau231 were the biomarkers with better performance in the literature [36,37].

Regarding screening approaches based on two-cut-offs [37], some studies used plasma p-Tau217 levels to separately analyze cognitively unimpaired and cognitively impaired individuals [39], or plasma p-Tau217 and p-Tau181 to classify subjects into Aβ+ and Aβ− [40]. These studies reported strong performance metrics in sensitivity (90%), specificity, and AUC (0.90). In the present study, the results were comparable, with the exception of sensitivity, which was slightly lower (67%). This could be explained by the inclusion of very early AD patients, even asymptomatic cases, as well as patients with MCI due to other causes. Nevertheless, this approach could be really useful as a diagnostic tool to identify patients who may benefit from recently approved disease-modifying treatments (e.g., lecanemab, donanemab) leading to rapid diagnosis and access to treatments, since in clinical practice, these therapies do not necessarily need to be restricted to patients in the very early stages of AD [41]. This advance will help screening patients because it offers significant advantages in terms of cost, reduced invasiveness, and large-scale capability. This investigation is demonstrated to be quite accurate and could help reduce the need for invasive and painful tests for the patients, optimizing the clinical workflow, ameliorating the benefit–risk ratio of some disease-modifying treatments such as lecanemab or donanemab. The implementation of such tools could help in the study and implementation of new therapies, reducing the necessity of more expensive methods also [42].

In the present work, both approaches (one-cut-off, two-cut-off) were compared. Overall, similar results were obtained in AUC (0.888 and 0.867, respectively), accuracy (77.4% and 77.1%, respectively) and PPV (96.5% and 100%). Although both methods demonstrated comparable performance, subtle differences were noted between them. The NPV and specificity were slightly higher, while the sensitivity was lower in the two-cut-off approach. Therefore, this is a suitable screening approach, since only 8% of the cases identified as non-AD were misclassified, and the uncertain cases reached 48% of the analyzed cases. On the other hand, the one-cut-off approach misclassified 19% of the identified non-AD cases and there is not an uncertain area. From this, the two-cut-off approach could be suitable for clinical implementation, representing a powerful validated screening tool.

In addition, the correlation study validates the shift toward plasma-based biomarkers for AD diagnosis, though CSF markers remain useful when ambiguous cases are detected. The integration of these findings with further advances is close to transforming AD diagnosis in the near future. It is important to highlight that plasma p-Tau 181 (CDR_GS rho = 0.362) and GFAP (MMSE rho = −0.354) seem to be powerful plasma biomarkers for clinical use, showing interesting correlations with several CSF biomarkers and promising indicators of neurocognitive decline. However, previous studies with plasma p-Tau217 demonstrate that this plasma biomarker is rising as a promising predictive variable to screen, diagnose, and differentiate AD from other neurodegenerative diseases with high accuracy [43,44,45].

The main limitation of the present study is the high cost of SIMOA^®^ technology analysis, as well as the requirement of specialized expertise, making it challenging to implement as standard diagnostic equipment in healthcare facilities. Nevertheless, the study has generated valuable insights about non-invasive biomarkers that could enhance the development of more transferable analytical methods [46]. Another limitation is the low sample size of the validation cohort in this study; so, further research is required in a large and external cohort in order to validate the potential diagnosis model in other clinical contexts. As key strengths, the diagnosis approach was validated in a temporal cohort, with participants representing the real-world data in a Cognitive Disorder Unit, as a key step in the clinical implementation in the hospital. Also, all the participants were characterized by CSF Aβ42/Aβ40 levels, allowing the development of a specific AD diagnosis approach.

## 5. Conclusions

A previous multivariate model based on plasma biomarkers (p-Tau181, GFAP, Aβ42/Aβ40) was validated using a temporal cohort of patients from the same cognitive disorder unit. To achieve this validation, two approaches (one-cut-off, two-cut-off) were evaluated, obtaining similar performance. Nevertheless, the two-cut-off approach showed lower discrepancy in identifying non-AD cases in comparison with the CSF biomarkers levels. These results highlight the potential as an effective screening tool for early AD, reducing the need for complementary tests (invasive and expensive) only in ambiguous cases. In general, the satisfactory validation obtained could be the first step for the clinical implementation of this AD screening method in a clinical unit.

## Figures and Tables

**Table 1:** Description of demographic and clinical characteristics of the participants groups.

		Non-AD (n = 19)	AD (n = 74)	*p* Value	r
Age (years) ^a^		69.00 (66.00–71.00)	71 (68.75–74.00)	0.025	−0.333
Gender (female) ^b^		8 (42.11)	44 (59.46)	0.174	
ApoE Genotype ^b^	ε3/ε3	15 (78.95)	34 (47.22)	0.011	
	ε2/ε3	3 (15.79)	3 (4.17)		
	ε2/ε4	0 (0.00)	4 (5.56)		
	ε3/ε4	1 (5.26)	28 (38.89)		
	ε4/ε4	0 (0.00)	3 (4.17)		
Educational level	Non-formal education	3 (15.79)	18 (24.32)	0.883	
	Primary education	6 (31.58)	21 (28.38)		
	Secondary education	4 (21.05)	13 (17.57)		
	University education	6 (31.58)	22 (29.73)		
CSF Aβ42 ^a^ (pg mL^−1^)		1279.00 (961.47–1524.00)	766.00 (599.00–924.00)	<0.001	0.666
CSF Aβ40 ^a^		12,854.00 (8811.00–14,831.50)	14,756.50 (12,112.50–17,514.25)	0.039	0.375
CSF Aβ42/Aβ40 ^a^		0.112 (0.101–0.115)	0.053 (0.045–0.059)	<0.001	1.000
CSF p-Tau181 ^a^ (pg mL^−1^)		37.00 (27.00–44.00)	89.00 (63.00–121.00)	<0.001	0.953
CSF t-Tau ^a^ (pg mL^−1^)		263.00 (164.00–313.00)	567.00 (439.50–762.50)	<0.001	0.808
CSF t-Tau/Aβ42 ^a^		0.23 (0.18–0.28)	0.77 (0.55–1.03)	<0.001	0.985
CSF NfL ^a^ (pg mL^−1^)		725.86 (460.79–869.50)	956.40 (794.00–1212.98)	0.001	0.584
AT classification ^b^	A+T+	0 (0.00)	44 (75.86)	<0.001	
	A-T-	19 (100.00)	0 (0.00)		
	A+T-	0 (0.00)	14 (24.14)		
	A-T+	0 (0.00)	0 (0.00)		
CDR sum of boxes ^a^ (score)		0.50 (0.00–3.00)	2.00 (1.00–3.00)	0.110	0.237
CDR_GS ^b^ (score)	0	7 (36.84)	10 (13.51)	0.005	
	0.5	10 (52.63)	63 (85.14)		
	1	2 (10.53)	1 (1.35)		
MMSE ^a^ (score)		27.00 (24.00–28.00)	26.00 (23.00–28.00)	0.407	0.123
RBANS_A ^a^ (score)		91.00 (76.50–97.75)	72.00 (60.00–88.00)	0.007	0.412
RBANS_L ^a^ (score)		89.00 (874.00–93.50)	79.00 (64.00–93.00)	0.167	0.210
RBANS_IM ^a^ (score)		85.00 (74.25–97.75)	69.00 (61.00–83.00)	0.003	0.460
RBANS_V/C ^a^ (score)		88.00 (71.25–98.25)	81.00 (71.25–92.00)	0.436	0.119
RBANS_DM ^a^ (score)		99.00 (87.50–103.50)	64.00 (55.00–86.25)	<0.001	0.610
GDS ^a^ (score)		17.00 (7.00–19.00)	9.00 (6.00–14.00)	0.048	0.298
ADCS-ADL-MCI ^a^ (score)		44.00 (38.00–47.00)	44.00 (39.00–47.00)	0.858	0.027
FAQ ^a^ (score)		1.00 (0.00–5.00)	3.50 (0.00–7.00)	0.077	0.287

^a^ Expressed as (median (IQR)); ^b^ expressed as (n (%)). IQR: Inter-quartile range. r: rank-biserial correlation r = 1 − (2U)/(n1 × n2) (from Mann–Whitney test). r, rank-biserial correlation (effect size): r < 0.3 small effect, 0.30 ≤ r < 0.50 moderate effect, r ≥ 0.50 large effect [34].

**Table 2:** Plasma biomarkers levels found in each participant’s group.

	Non-AD (n = 19)	AD (n = 74)	*p* Value	r
Plasma p-Tau181 ^a^ (pg mL^−1^)	13.00 (9.00–17.00)	29.00 (20.00–37.00)	<0.001	0.733
Plasma Aβ42/Aβ40 ^a^	0.042 (0.038–0.047)	0.039 (0.036–0.043)	0.046	0.297
Plasma GFAP ^a^ (pg mL^−1^)	115.00 (78.00–185.00)	194.50 (140.75–280.50)	<0.001	0.481

^a^ Expressed as (median (IQR)). IQR: Inter-quartile range. r: rank-biserial correlation r = 1 − (2U)/(n1 × n2) (from Mann–Whitney test). r, rank-biserial correlation (effect size): r < 0.3 small effect, 0.30 ≤ r < 0.50 moderate effect, r ≥ 0.50 large effect [34].

**Table 3:** Correlations between plasma biomarkers levels and clinical variables (CSF biomarkers and neurophyscological tests) by means of Spearman test (* *p* < 0.05).

	Plasma p-Tau181 (pg mL^−1^)	Plasma Aβ42/Aβ40 (pg mL^−1^)	Plasma GFAP (pg mL^−1^)
CDR_GS (score)	0.362 * (*p* < 0.001)	−0.256 * (0.013)	0.324 * (0.002)
CDR sum of boxes (score)	0.354 * (0.001)	−0.283 * (0.006)	0.330 * (0.001)
MMSE (score)	−0.280 * (0.007)	0.219 * (0.035)	−0.354 * (0.001)
RBANS_MI (score)	−0.325 * (0.002)	0.134 (0.202)	−0.265 * (0.011)
RBANS_VC (score)	−0.221 * (0.035)	0.128 (0.224)	−0.165 (0.116)
RBANS_L (score)	−0.205 (0.052)	0.219 * (0.036)	−0.329 * (0.001)
RBANS_A (score)	−0.174 (0.099)	0.139 (0.187)	−0.224 * (0.032)
RBANS_MR (score)	−0.455 * (*p* < 0.001)	0.296 * (0.004)	−0.402 * (*p* < 0.001)
FAQ (score)	0.271 * (0.013)	−0.325 * (0.002)	0.204 (0.061)
ADCS-ADL-MCI (score)	−0.109 (0.303)	0.047 (0.654)	−0.089 (0.399)
GDS (score)	−0.225 * (0.031)	0.163 (0.119)	−0.137 (0.190)
CSF Aβ42 (pg mL-1)	−0.390 * (0.001)	0.108 (0.353)	−0.091 (0.434)
CSF t-Tau (pg mL^−1^)	0.493 * (*p* < 0.001)	−0.256 * (0.021)	0.402 * (*p* < 0.001)
CSF p-Tau181 (pg mL^−1^)	0.582 * (*p* < 0.001)	−0.314 * (0.006)	0.448 * (*p* < 0.001)
CSF Aβ40 (pg mL^−1^)	0.162 (0.213)	−0.149 (0.251)	0.208 (0.108)
CSF Aβ42/Aβ40	−0.500 * (*p* < 0.001)	0.286 * (0.025)	−0.275 * (0.032)
CSF NfL (pg mL^−1^)	0.258 * (0.047)	−0.487 * (*p* < 0.001)	0.399 * (0.002)
CSF t-Tau/Aβ42	0.597 (*p* < 0.001)	−0.303 * (0.008)	0.360 * (0.001)

Data are expressed as rank correlation coefficients (rho) (*p* value).

**Table 4:** Performance results from the temporal validation of both AD diagnosis approaches.

Diagnosis Index	One-Cut-Off Approach	Two-Cut-Off Approach
Cut-off values	0.5	0.41–0.66
AUC	0.888	0.867
PPV (CI 95%)	96.5% (88.1−99.0%)	100.0% (85.1–100%)
NPV (CI 95%)	47.2% (32.0–63.0%)	57.7% (38.9–74.5%)
Sensitivity (CI 95%)	74.3% (63.3–82.9%)	66.7% (49.6–80.2%)
Specificity (CI 95%)	89.5% (68.6–97.1%)	99.9% (79.5–100%)
Accuracy (%)	77.4% (67.9–84.7%)	77.1% (63.4–86.7%)

CI: Confidence interval; AUC: Area under the curve; NPV: Negative predictive value; PPV: Positive predictive value.

## Data Availability

The raw data supporting the conclusions of this article will be made available by the authors on request.

## List of Abbreviations

AD	Alzheimer’s Disease
Aβ	Amyloid-beta
GFAP	Glial fibrillary acidic protein
CSF	Cerebrospinal fluid
PET	Positron Emission Tomography
t-Tau	Total-Tau
p-Tau	Phosphorylated Tau
NfL	Neurofilament light
ADNI	Alzheimer’s Disease Neuroimaging Initiative
ApoE	Apolipoprotein E
BIN1	Bridging integrator 1
CD2AP	CD2 associated protein
CLU	Clusterin
INPP5D	Inositol polyphosphate-5-phosphatase D
MCI	Mild Cognitive Impairment
MACC	Memory Aging and Cognition Centre
MAP	Memory & Aging Program
AUC	Area Under the Curve
CDR	Clinical Dementia Rating
MMSE	Mini-Mental State Examination
RBANS	Repeatable Battery for the Assessment of Neuropsychological Status
FAQ	Functional Activities Questionnaire
ADCS-ADL	Alzheimer’s Disease Cooperative Study—Activities of Daily Living
GDS	Geriatric Depression Scale
PPV	Positive predictive value
NPV	Negative predictive value
IQR	Inter-quartile range
CI	Confidence interval
